# Immune Evasion Strategies of *Trypanosoma brucei* within the Mammalian Host: Progression to Pathogenicity

**DOI:** 10.3389/fimmu.2016.00233

**Published:** 2016-06-24

**Authors:** Benoît Stijlemans, Guy Caljon, Jan Van Den Abbeele, Jo A. Van Ginderachter, Stefan Magez, Carl De Trez

**Affiliations:** ^1^Laboratory of Myeloid Cell Immunology, VIB Inflammation Research Center, Ghent, Belgium; ^2^Laboratory of Cellular and Molecular Immunology, Vrije Universiteit Brussel (VUB), Brussels, Belgium; ^3^Laboratory for Microbiology, Parasitology and Hygiene (LMPH), University of Antwerp, Wilrijk, Belgium; ^4^Unit of Veterinary Protozoology, Department of Biomedical Sciences, Institute of Tropical Medicine Antwerp (ITM), Antwerp, Belgium; ^5^Department of Structural Biology, VIB, Brussels, Belgium

**Keywords:** African trypanosomosis, *T. brucei*, tsetse fly, innate immune response, pathogenicity

## Abstract

The diseases caused by African trypanosomes (AT) are of both medical and veterinary importance and have adversely influenced the economic development of sub-Saharan Africa. Moreover, so far not a single field applicable vaccine exists, and chemotherapy is the only strategy available to treat the disease. These strictly extracellular protozoan parasites are confronted with different arms of the host’s immune response (cellular as well as humoral) and via an elaborate and efficient (vector)–parasite–host interplay they have evolved efficient immune escape mechanisms to evade/manipulate the entire host immune response. This is of importance, since these parasites need to survive sufficiently long in their mammalian/vector host in order to complete their life cycle/transmission. Here, we will give an overview of the different mechanisms AT (i.e. *T. brucei* as a model organism) employ, comprising both tsetse fly saliva and parasite-derived components to modulate host innate immune responses thereby sculpturing an environment that allows survival and development within the mammalian host.

## Introduction

Trypanosomatids, which include African trypanosomes (AT), American trypanosomes (i.e. *Trypanosoma cruzi* causing Chagas’ disease) and different *Leishmania* species, comprise a large group of flagellated unicellular protozoa with a parasitic and complex digenetic life cycle. These diseases, exhibiting high morbidity and mortality rates, affect millions of impoverished populations in the developing world, display a limited response to chemotherapy, and are classified as neglected tropical diseases by the World Health Organization (WHO) (1, 2). In contrast to the other two trypanosomatids, the diseases caused by AT are of both medical and veterinary importance and adversely influence the economic development of sub-Saharan Africa. Indeed, upon transmission through the bite of their blood-feeding vector (i.e., the tsetse fly, *Glossina spp*.), these parasites can cause fatal diseases in mammals, commonly called sleeping sickness in humans [Human African Trypanosomosis (HAT)] or Nagana (AAT, Animal African Trypanosomosis) in domestic livestock. According to the WHO, from the 60 million people living in the risk areas (i.e., the “tsetse” belt), approximately 300,000 people are currently infected with trypanosomes leading to 10,000–40,000 deaths annually (3, 4). The human pathogens *Trypanosoma brucei gambiense* (accounting for over 95% of cases) and *Trypanosoma brucei rhodesiense* (accounting for the remainder of cases) do not only differ in geographical distribution but also differ biologically, clinically, therapeutically, and epidemiologically and cause separate diseases (3, 5, 6). By contrast, the animal pathogens causing either Nagana (*Trypanosoma brucei brucei, Trypanosoma congolense, Trypanosoma vivax*) or Surra (*Trypanosoma evansi*) or Dourine (*Trypanosoma equiperdum*), do not cause disease in humans. Of note, some atypical human infections with animal trypanosomes, such as *T. evansi*, have been reported, which relate to deficiencies in the innate resistance to these otherwise non-human pathogens (7). Yet, AAT mainly caused by *T. congolense* and to a lesser extent by *T. b. brucei* and *T. vivax* forms a major constraint on cattle production. Hence, Nagana has a great impact on the nutrition of millions of people living in the most endemic areas, and on the agriculture economics of their countries, resulting in an estimated annual economic cost of about US$ 4 billion (8). Furthermore, the lack of prospect for vaccine development against AT is reinforced by (i) the fact that pharmaceutical companies are less prone to engage/invest in drug discovery/development against diseases that affect the poorest people, (ii) the political instability of the affected regions, (iii) the fact that wild animals function as reservoir of the parasite and, therefore, hamper the control of the disease, and (iv) the inappropriate use of the available drugs resulting in the emergence of drug resistance (8–11). Nevertheless, so far chemotherapy using compounds that target unique organelles of trypanosomes (i.e., glycosomes and kinetoplast) that are absent in the mammalian host and/or trypanosome metabolic pathways that differ from the host counterparts (carbohydrate metabolism, protein and lipid modifications, programed cell death) remain the only therapeutic choice for these diseases (12–16).

In contrast to the other two trypanosomatids, AT are strictly extracellular. Hence, they have developed efficient immune escape mechanisms to evade/manipulate the entire host immune response (cellular and humoral), involving an elaborate and efficient vector–parasite–host interplay, to survive sufficiently long in their mammalian host in order to complete their life cycle/transmission. Most of the research on AT uses murine models, which are more easily amenable compared to cattle or other domestic animals. Furthermore, given that the HAT causing *T. b. rhodesiense* and *T. b. gambiense* parasites highly resemble *T. b. brucei* (a non-human pathogenic subspecies causing Nagana), and chronic murine HAT models are scarce, the majority of research uses *T. b. brucei* as a model (17, 18). In this review, we will give an overview of the immunological events occurring during the early stages of infection within the mammalian host, using *T. b. brucei* as a model organism. We will also describe the different strategies that trypanosomes developed to sequentially activate and modulate innate immune responses to successfully escape immune elimination and maintain a chronic infection. Finally, we will discuss briefly how the host innate/adaptive immune response can culminate in immunopathogenicity development in trypanosusceptible animals.

## Evasion Mechanisms of African Trypanosomes in the Mammalian Host

*Trypanosoma brucei* parasites cycle between the alimentary tract/salivary glands of the tsetse fly vector and the blood/tissues of the mammalian host. In each host, parasites undergo many life cycle changes (i.e., in the tsetse fly as procyclic/epimastigote/metacyclic forms and in the mammalian host as bloodstream forms) with discrete/important morphological and metabolic changes, which are programed precisely to adapt to different growth conditions/nutrient availability imposed by the different hosts and microenvironments they inhabit (19–22). These include, fine-tuning of energy metabolism, organelle reorganization, and biochemical and structural remodeling, which is supported by major changes in gene expression and proliferation status to adapt/survive in the different hosts (23). Furthermore, within the mammalian host, the complex life cycle of *T. brucei* consists of a succession of proliferative [long slender (LS)] and quiescent [short stumpy (SS)] developmental forms, which vary in cell architecture and function (23). Hereby, in response to a quorum sensing mechanism involving a stumpy-inducing factor (SIF) (24, 25), the LS forms differentiate into SS forms that are pre-adapted for the next developmental transition to procyclic forms, which occurs after ingestion by a tsetse fly (26).

Due to millions of years of co-evolution, these parasites have been able to thwart host innate responses and escape early recognition, allowing the initiation of infection in their respective hosts. In this section, we will give an overview of how trypanosomes can benefit from tsetse fly saliva components to initiate infection and subsequently how trypanosomal components can dampen/sculpture distinct innate immune responses in the mammalian host, which are pivotal in allowing early parasite infection and subsequent chronic infection.

### Tsetse Fly Saliva Components Sculpture an Immune-Tolerant Microenvironment to Allow Establishment of Trypanosome Infections

**Figure 1:**
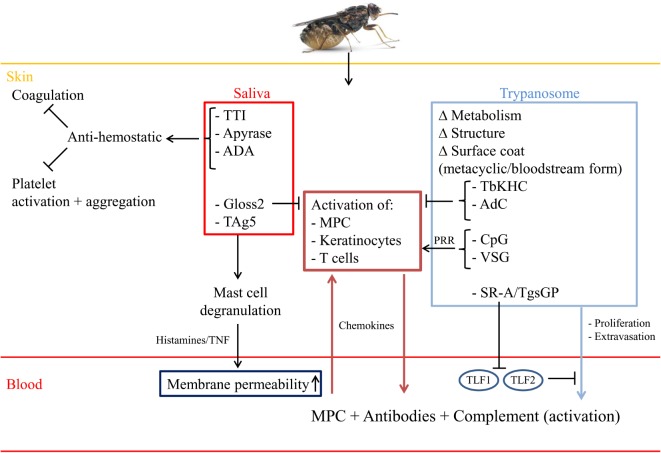
**Saliva components and parasite-derived factors sculpture the skin microenvironment**. Upon the bite of a trypanosome-infected tsetse fly, trypanosomes and saliva components are inoculated intradermally leading to modulation of the skin microenvironment into a trypanosome receptive habitat. To this end, saliva components, such as TTI, a 5′Nucleotidase-related apyrase and Adenosine Deaminase-related proteins (ADA) prevent blood coagulation and platelet activation/aggregation, while the TAg5 allergen leads to activation of mast cells. Subsequently, these mast cells degranulate and release histamine and TNF, thereby increasing vasodilatation and allowing extra/intravasation of immune cells [myeloid phagocytic cells (MPC)] as well as parasites. In addition, this will allow infiltration of antibodies as well as complement factors needed for early parasite elimination. Yet, also the complement system (via C3a and C5a) can contribute to (i) increased vasopermeability and (ii) recruitment and activation of immune cells (PMN,…). By contrast, the Gloss2 peptide is able to downregulate inflammatory responses that are triggered upon breaching the skin anatomical barrier and/or encounter of metacyclic trypanosomes. Within the skin, these metacyclic parasites transform into LS bloodstream forms, which is associated with metabolic/structural/morphological changes, including switching of their metacyclic VSG into a blood-stream form VSG, required for survival within the mammalian host. The PAMPs of these pathogens (such as VSG and CpG) can be recognized by tissue-resident MPC or keratinocytes expressing PRR, leading to their activation and subsequent release of innate immune response triggering signals. For instance, release of chemokines will trigger the recruitment of MPC, which can amplify the immune response needed to eliminate skin-associated trypanosomes. Yet, trypanosomes try to dampen the initial pro-inflammatory immune response by (i) releasing TbKHC or (ii) following phagocytosis of altruistic parasites releasing TbAdC, thereby allowing the remaining parasite to survive and proliferate. Within the blood circulation, the parasites encounter the trypanolytic molecules TLF-1 and 2, leading to elimination of non-primate infecting parasites. Yet, HAT-causing parasites express SRA or TgsGP, which inactivate the (ApoL1/HpR) TLF-1/2-mediated trypanolytic effects, thereby allowing proliferation within the blood circulation.

A typical infection in the mammalian host begins when the infective stage, i.e., the metacyclic form, is co-injected with saliva intradermally by the tsetse fly. Hereby, the skin of the vertebrate host is a crucial anatomical barrier that pathogens have to overcome in order to establish infection. Within this microenvironment, pharmacological as well as immunological processes occur aimed at preventing pathogen development, whereby cells (lymphocytes, myeloid phagocytes, keratinocytes,…) sense the presence of damage-associated molecular patterns (DAMPs) as well as pathogen-associated molecular patterns (PAMPs) via different pattern recognition receptors (PRRs), leading to the secretion of pro-inflammatory cytokines, type-I IFN, chemokines, reactive oxygen and nitrogen species, and antimicrobial peptides (27–29). Yet, during evolution, the skin has become a key interface for arthropod-borne diseases, whereby the pathogen in concert with saliva components transforms the skin barrier into an immune-tolerant organ supporting parasite development (30–32). This was strengthened by the observations by Caljon et al. (33) that the presence of tsetse fly saliva allowed a faster onset of the disease, which was associated with a reduced induction of inflammatory mediators at the site of infection and the interference of tsetse fly saliva with host hemostatic reactions (34). Indeed, tsetse fly saliva was shown to exert a dual pharmacological role (see Figure 1, red panel), (i) interfere with vertebrate host responses to enable successful blood feeding via the suppression of vasoconstriction, platelet aggregation, and coagulation [involving the anti-coagulant thrombin inhibitor (TTI), a 5′Nucleotidase-related apyrase and Adenosine Deaminase-related proteins (ADA)] (35–41) and (ii) modulate the host immune environment at the bite site that impacts pathogen transmission (42–44). Saliva was also reported to be highly immunogenic/allergenic in nature, thereby promoting infection onset in saliva immunized animals (45). For instance, Tsetse Antigen5 (TAg5) was shown to sensitize mice and trigger acute hypersensitivity reactions, which in turn could allow more efficient parasite extravasation into the blood circulation (46). A recently identified immunoregulatory peptide Gloss2 in tsetse fly saliva was shown to inhibit the secretion of trypanolytic molecules, such as TNF, and other pro-inflammatory cytokines, such as IFN-γ and IL-6, which could allow parasites to avoid initial elimination (44). Yet, through the use of transcriptome analyses and the availability of a partially annotated tsetse genome, it might be expected that many more proteins will be identified in the near future (34, 47).

Within the local immune-tolerant skin microenvironment, the metacyclic parasites respond to the increased temperature and rapidly transform into blood-stage trypomastigotes (LS forms), which divide by binary fission in the interstitial spaces at the bite site. Subsequently, they disseminate via the draining lymph nodes (48, 49). The first visible sign of a trypanosome infection coincides in many but not all instances with the occurrence of a “chancre,” several days after infection (48, 50, 51). This development (onset, size, and duration) of the chancre correlates with the number of metacyclic parasites inoculated into the skin and is due to a local immune response directed against the variable antigen type (VAT) of the proliferating metacyclic forms (48). This consists of buildup of metabolic waste and cell debris from apoptotic cells, mainly neutrophils, releasing their intracellular cargo [i.e., neutrophil extracellular traps (NETs), antimicrobial peptides] aiming at capturing and subsequently killing the pathogens (52). Of note, although there are so far no reports documenting the contribution of neutrophils at the early stages of AT infection, the contribution of these phagocytes are documented in many other protozoan infections, such as Leishmaniasis and Malaria (52–54). Subsequently, the apoptotic cells in concert with parasite- and vector-derived components will be internalized by myeloid phagocytic cells (MPC) and degraded/processed to initiate innate immune responses (52–54). Also CD4^+^ T lymphocytes were shown to play a key role in chancre formation, since *in vivo* depletion of CD4^+^ T cells before inoculation of trypanosomes via a tsetse-fly bite resulted in a significant reduction of chancre formation (50).

### Trypanosome-Derived Components Allow Parasite Survival and Sculpture Host Responses

#### SRA and TgsGP Allow Resistance to Normal Human Serum-Mediated Lysis

An important first step in the initiation and establishment of a trypanosome infection in the mammalian host is associated with cell cycle re-entry and metabolic/morphological/structural changes (see Figure 1, blue panel). This is required for acquisition of nutrients (i.e., glucose/iron/heme) in order to proliferate and subsequently activate immune evasion mechanisms to establish infection (20, 48, 55). Since trypanosomes are deficient in heme biosynthesis and heme cannot diffuse through the parasites’ membrane (56–58), they require uptake of exogenous heme by the haptoglobin (Hp)–hemoglobin (Hb) receptor (HpHbR) located in the parasites’ flagellar pocket (59). Following release of Hb from destroyed erythrocytes, it will be complexed with Hp, forming a Hp–Hb complex, which can be recognized by the myeloid phagocyte system (MPS) via CD163 and by the trypanosomal HpHbR. This will allow parasites to acquire and incorporate heme into intracellular hemoproteins required for optimal parasite growth and resistance to the oxidative burst by host cells. However, the HpHbR is also involved in primate innate immunity against certain trypanosome species (60, 61). Indeed, the serum of catarrhine primates and humans contains two trypanolytic particles: (i) a 500 kDa high-density lipoprotein (HDL)-bound trypanosome lytic factor (TLF)-1 and (ii) a 2 mDa large lipid-poor (<2%) IgM/apolipoprotein A-1 complex called TLF-2, that harbor the trypanolytic primate-specific apolipoprotein L1 (ApoL1), ApoA1, and Hp-related protein (Hpr) (62–65). Importantly, Hpr is a gene duplication product exhibiting high homology with Hp, which interacts with Hb to form an Hpr–Hb complex on the TLF-1 particles (66, 67). Following binding of the TLF-1 particle to the HpHbR (60, 68, 69), the entire TLF-1 particle is endocytosed and targeted to the lysosome. Subsequently, ApoL1 forms a pore in the endolysosomal membrane and triggers lysosomal swelling leading to the lethal release of lysosomal content into the parasites’ cytosol (70–76). In addition, it was shown that the C-terminal kinesin *TbKIFC1* is involved in ApoL1-mediated lysis, whereby it transports ApoL1 from the endolysosomal membrane to the mitochondrion, leading to mitochondrial membrane depolarization and fenestration and subsequently lysis (77). Two different models are proposed to explain TLF-2 mediated killing; (i) since both Hpr and ApoL1 are present in this particle and TLF-2 killing of *T. b. brucei* is partly dependent on the *Tb*HpHbR receptor for uptake, TLF-2 may function in a manner similar to TLF-1 (60, 69). Yet, given that TLF-2 killing was not inhibited by the addition of Hp, a potent competitive inhibitor of TLF-1 uptake, it is more likely that TLF-2 has a different mode of internalization than TLF-1 (62, 73). (ii) TLF-2 uptake may also be linked to ApoL1 interaction with the *T. b. brucei* variable surface glycoprotein (VSG) coat or TLF-2-associated IgM may bind *T. b. brucei*, as it is the only protein component that distinguishes both classes of TLF (78). Yet, so far no results supporting either mechanism of TLF-2 binding to *T. b. brucei* have been reported.

In contrast to the widespread *T. b. brucei* subspecies, which is highly infectious in many non-primate species that do not express Hpr and ApoL1 (79), the human pathogenic subspecies *T. b. rhodesiense* and *T. b. gambiense* express resistance proteins. Indeed, *T. b. rhodesiense* expresses a serum resistance antigen (SRA) and *T. b. gambiense* expresses a specific glycoprotein (TgsGP) counteracting ApoL1 activity (80–82), thereby enabling these parasites to evade the lethal action of TLF particles (see Figure 1, blue panel). Furthermore, *T. b. gambiense* exhibits low-level HpHbR expression and harbors an amino acid substitution (L210S) in HpHbR, leading to reduced TLF-1 uptake (76, 80–85). Recently, it was shown that SRA can be transferred from *T. b. rhodesiense* to *T. b. brucei* by membranous nanotubes that originate from the flagellar membrane and disassociate into free extracellular vesicles (EV) (86). Hence, this could result in the exchange of virulence factors that confer resistance to innate elimination.

#### *T. brucei*-Derived Kinesin Heavy Chain (TbKHC1) and Adenylate Cyclase Dampen Inflammation and Promote Parasite Growth

Besides parasite-derived factors playing a role in resistance to normal human serum (NHS), some parasite-derived molecules (see Figure 1, blue panel) are also able to dampen pro-inflammatory responses (TNF, NO) by classically activated macrophages (M1), needed for initial parasite control. One such important *T. brucei* protein is the Kinesin Heavy Chain 1 (TbKHC1) (87), which is released by the parasites in the environment via an unknown mechanism and sustains the development of the first (most prominent) peak of parasitemia in the blood and its control by the host. Following binding of TbKHC1 to the SIGN-R1 molecule (i.e., a surface C-type lectin expressed mainly by marginal zone macrophages within the spleen), the arginine/NO metabolism is modulated in favor of arginase activity via an IL-10-dependent induction of arginase-1 and down-regulation of iNOS activities. In turn, this stimulates the production by the host of l-ornithine and hereby the synthesis of polyamines, which are essential nutrients for growth of trypanosomes in the host (88). Consequently, IL-10/arginase-1-producing immune cells are impaired in their capacity to destroy the parasite, thereby favoring parasite settlement. Another factor that trypanosomes use to establish infection comprises in the large family of transmembrane receptor-like adenylate cyclases (AdCs), called *T. brucei* Adenylate Cyclase (TbAdC) (89), which converts ATP into cyclic adenosine monophosphate (cAMP). During steady-state conditions, the TbAdC levels are low as is the cAMP production, yet upon stress (such as phagocytosis by M1 cells) the TbAdC levels can be elevated ~250-fold above the basal cellular content (89, 90). Subsequently, the cytoplasmic cAMP levels within the phagocytes increase, activating protein kinase A and leading to the inhibition of the synthesis of the trypanolytic cytokine TNF (91, 92). Hence, it seems that trypanosomes have developed a system whereby altruistic parasites are phagocytosed, thereby disabling the M1-mediated innate immune response required for parasite control (see Figure 1), and paving the way for initiation and establishment of the first wave of parasitemia.

#### Surface Coat Remodeling Prevents Elimination by the Humoral Immune Response

Given that AT are strictly extracellular parasites, they are confronted with the hosts’ humoral immune response. Yet, one of the most fundamental changes occurring when parasites are inoculated into the mammalian host is the remodeling of the parasite cell surface (93). Indeed, within the mammalian host the metacyclic forms rapidly transform into the typical LS bloodstream forms expressing a different uniform VSG coat (94). This VSG coat consists of 5 × 10^6^ homodimers of 50–60 kDa subunits held on the extracellular face of the plasma membrane by a glycosylphosphatidylinositol (GPI) anchor (95), which consists of a ethanolaminephosphate-6-mannose-α1,2-mannose-α1,6-mannose-α1,4-glucosamine-α1,6-myo-inositol-1-phospholipid motif and a short galactose chain (96–99). Despite great variations in primary sequence, the secondary and tertiary structural features are highly conserved within the ordered coat structure (100). Although VSG molecules are free to diffuse in the plane of the membrane (101, 102), this ~15-nm-thick VSG coat has a dual role: shield off buried invariant proteins from recognition by the hosts’ innate/acquired immune system and protect bloodstream parasites against complement-mediated lysis. Indeed, activation of the alternative pathway, which occurs in the absence of specific antibodies (Abs), may potentially play a crucial role in parasite clearance during the early stage of infection. Yet, it was shown by Devine et al. (103) that *T. b. gambiense* parasites, which are covered by C3, specifically inhibit the activation of the alternative pathway through their VSGs by masking sites on the plasma membrane, which are capable of promoting alternative pathway activation (104). Hence, the activation of the alternative pathway did not proceed further than the establishment of the C3 convertase, thereby impairing the generation of the terminal complex (C5–C9) which normally induces trypanolysis (103). In addition, soluble complement molecules, such as C3a and C5a, secreted during early stages of trypanosome infection, can further contribute to the initiation of the early inflammatory immune response within the chancre and may also act as (i) chemotactic agents attracting phagocytes to the site of infection and (ii) release histamine from mast cells thereby increasing microvascular permeability (105), which would allow/enable parasite extravasation into the blood circulation. Of note, the classical pathway, activated by immune complexes of trypanosome antigens and Abs, seems to contribute to trypanosome clearance through antibody-mediated trypanolysis and/or phagocytosis, which is of importance during peak parasitemia clearance (see later). Yet, also in this scenario, parasites are able to eliminate/remove surface-bound IgG (immune complexes) as well as complement through their rapid VSG recycling system and thereby prevent elimination (106). Furthermore, since complement is essential in antibody-mediated destruction of trypanosomes, by releasing vast amounts of soluble VSG (sVSG), mainly observed at the peak of parasitemia, this will scavenge complement factors and, hence, induce a state of hypocomplementemia (107, 108). This might favor the survival and escape of a minority of the parasites.

Additionally, binding of anti-VSG IgG or IgM to the trypanosome’s coat results in parasite aggregation. Yet, trypanosomes are able to disaggregate in an energy-dependent manner involving protein kinase-C as part of the defense against the host humoral immune system (109). Hence, this could function as a survival strategy in the presence of antibody prior to the occurrence of VSG switching (109). The parasite’s surface consisting of repetitive monotypic VSG molecules can cross-link B cell receptors (BCRs) and subsequently lead to T-cell-independent B-cell activation (110). However, during the process of antigenic variation (from metacyclic form toward trypomastigote form) mediated via changing VSG expression sites (i.e., *in situ* switching or transcriptional control) or by gene replacement resulting in a switch of the terminal telomeric VSG gene, heterologous VSG molecules are presented on the surface, thereby forming a mosaic VSG coat, which prevents direct B cell activation until a VSG uniformity is obtained (111, 112). This in turn might allow parasites to transiently escape T cell-independent B cell-mediated elimination and gives time to transform into trypomastigote forms adapted to survive in the mammalian host. Hence, this process gives the parasites an immunological advantage during the process of antigenic variation and is an efficient mechanism to escape antibody-mediated elimination during the early as well as chronic stage of infection (111).

#### VSG and VSG-Derived Fragments Trigger Different Cellular Innate Immune Responses

**Figure 2:**
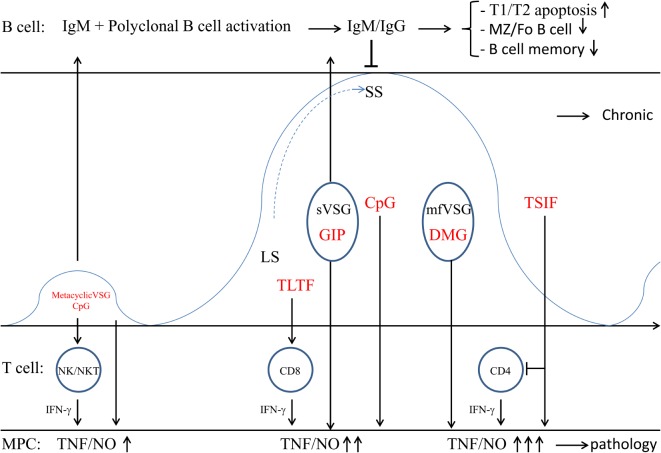
**Trypanosome establishment within the mammalian host**. Within the blood circulation (several days post infection) the metacyclic trypanosomes give rise to a first small peak (which is not always observed). Subsequently, the metacyclic trypanosomes change their metacyclic VSG coat into the bloodstream VSG, thereby expressing a mosaic VSG that prevents Ab-mediated elimination. This dense VSG coat also prevents recognition of buried epitopes, including binding of complement factors (C3) to their surface. Also Ab-mediated elimination is prevented due to the rapid recycling of these VSG–Ab complexes and VSG shedding (i.e., sVSG release) that in turn scavenges circulating complement. Recognition of sVSG via SR-A on myeloid cells, in concert with CpG recognized via TLR9, results in the activation of MPC, which trigger activation of NK/NKT and T cells. In turn, these cells produce IFN-γ needed for proper activation of myeloid cells (M1 cells) and subsequent release of pro-inflammatory mediators (TNF/NO). Of note, initially, when GIP-sVSG is released via PLC activation due to stress prior to IFN-γ production, there is a weak activation of myeloid cells. Yet, triggering of PRR at the level of B cells (i.e., TLR9 via CpG) can also lead to polyclonal B-cell activation. Subsequently, parasites rapidly multiply as LS forms giving rise to the most prominent parasitemia peak. Trypanosomes also release TLTF that triggers IFN-γ production by CD8^+^ T cells, which in turn stimulates parasite proliferation. However, IFN-γ exposure in concert with GIP-sVSG release will trigger an enhanced production of trypanolytic molecules by myeloid cells, which in concert with anti-VSG antibodies are needed for peak parasitemia control. Upon reaching the peak of parasitemia, the majority of the parasites differentiate into non-proliferative SS forms that are pre-adapted for uptake by tsetse flies, while a minority undergoes antigenic variation. Yet, in the mammalian host, these SS forms are deemed to die, thereby releasing mfVSG as well as CpG. These molecules exert dual functions; (i) the DMG of mfVSG triggers macrophage hyperactivation and LPS-hypersensitivity, while CpG further fuels polyclonal B-cell activation. These B cells can differentiate into short-lived plasmablasts (producing unspecific IgM) and ultimately results in apoptosis/elimination of all B-cell subsets and loss of B-cell memory. At this stage of infection, parasites also release TSIF that further stimulates the production of suppressive M1 and induces T-cell suppression. Once the first peak of parasitemia is controlled, the infection is established and the hosts’ adaptive immune response will develop, whereby the B- and T-cell response are impaired and there is a polarized M1 activation leading the trypanosomosis-associated pathogenicity.

The VSG coat plays a key role in the interaction with the host, whereby it is involved in a population survival strategy through antigenic variation as well as in an individual cell survival strategy through rapid endocytosis, removal of bound antibody, and recycling back to the cell surface (106). The parasites not only use the VSG as an efficient escape mechanism jeopardizing the induction of an effective antibody response (113–115), but also use it as means to modulate the hosts’ cellular responses. Indeed, *T. brucei* parasites contain an endogenous phospholipase C (PLC) known as the GPI-PLC, which is activated upon hypotonic lysis, stress, or during antigenic variation (90, 116, 117), and shown not to be essential but rather to act as a virulence factor given that a PLC^−/−^ mutant was attenuated in mice (118). Activation of the GPI-PLC hydrolyzes the GPI-anchor on the VSG (119, 120). This hydrolysis will convert the hydrophobic membrane-form VSG (mfVSG) into a water sVSG (117), thereby leaving the dimyristoyl glycerol (DMG) compound of the GPI-anchor in the membrane and releasing the glycosylinositolphosphate (GIP)-VSG part (121). Both components (DMG and GIP-VSG) exhibit distinct functions as far as activating potential of host immune cells is concerned (121, 122). Indeed, the GIP-VSG moiety is recognized by a Type A scavenger receptor expressed on myeloid cells, thereby initiating the activation of NF-κB and MAPK pathways and the expression of pro-inflammatory genes, such as TNF-α, IL-6, IL-12p40, and GM-CSF (123). This is further amplified when myeloid cells are primed with T-cell derived IFN-γ (124). Hereby, the galactose side chain of VSG is responsible for TNF-α production following activation of the protein tyrosine kinase (PTK) pathway (121, 125). However, reversing the order of exposure (i.e., exposing myeloid cells to GIP-VSG before IFN-γ stimulation) resulted in a down-regulation of IFN-γ-inducible responses, including transcription of inducible NO synthase and secretion of NO, which was associated with reduction in the level of STAT1 phosphorylation (126). This event might be of importance during the initial stage of infection, i.e., when sVSG is released from metacyclic forms during the early transition into bloodstream forms (see Figure 2). The GPI moiety, and in particular its DMG anchor that is released mainly during the descending phase of acute infection and during chronic infection, activates the protein kinase-C (PKC) pathway, and mediates macrophage priming/hyperactivation and LPS hyper-responsiveness in a MyD88-dependent manner (121, 122, 127, 128). Importantly, also in experimental bovine models, the DMG compound was shown to be crucial for M1 over-activation (129). In addition, the DMG compound of the mfVSG anchor seems to be crucial, via its IL-1α-inducing and -priming activity, in further fueling TNF induction (130).

#### CpG DNA is Used as Immunomodulatory Molecule to Trigger Macrophage Activation and Early Polyclonal B Cell Activation

Non-mammalian genomic DNA (i.e., CpG DNA) can also induce a host immune response (131). In this context, low amounts of tsetse-inoculated metacyclic parasites or SS blood-stream form parasites, continuously generated during the parasite cycle in the mammalian host, are eliminated/lysed giving rise to release of CpG DNA into circulation (see Figure 2). In turn, these CpG oligonucleotides trigger TLR9 signaling leading to the induction of M1 activation and polyclonal B-cell activation and subsequent isotype switching (132, 133). Importantly, CpG-mediated signaling can independently or synergistically with parasite-derived lipid or protein molecules (see further) activate the production of pro-inflammatory cytokines and NO needed for optimal peak parasitemia control. Indeed, as shown by Drennan et al. (127), during *T. brucei* infections, there is partial requirement for TLR9 signaling in the production of IFN-γ and VSG-specific IgG2a antibodies and for mammalian TLR family and MyD88 signaling in the innate immune recognition of *T. brucei*. Polyclonal B-cell activation, on the other hand, which is induced independently of BCR specificity, may play an important role in the defense against infections by enhancing natural antibody production and inducing memory B cells. Hence, polyclonal B cell activation increases the levels of natural antibodies to keep up with multiplication of the microorganisms, thus containing pathogen dissemination. Although triggering of polyclonal B cell activation is a natural innate immune response induced by many pathogens, the induction of polyclonal B cell activation (B cell expansion) might also be used as an immune evasion mechanism, whereby unselectively differentiating B cells can differentiate into short-lived plasmablasts (producing unspecific IgM), which ultimately results in apoptosis/elimination of the targeted B cell population (134, 135). In addition, regulatory B cells might also be induced and exert an immunosuppressive function by the secretion of IL-10, IL-17, IL-35, and transforming growth factor-β (TGFβ), and thereby dampen the initial pro-inflammatory immune response aimed at controlling infection (110, 136). However, so far no evidence of the occurrence of regulatory B cells is provided in this model.

#### TLTF Triggers IFN-γ Production by CD8^+^ T Cells

Another trypanosome-derived factor documented to play a key role in early parasite–host interactions is the trypanosome-derived lymphocyte-triggering factor (TLTF), a secreted 185 kDa invariant glycoprotein able to trigger IFN-γ production by CD8^+^ T cells (137–140). It was shown by Hamadien et al. (138) that early during *T. brucei* infection (day 3 p.i.) high levels of TLTF could be measured in the serum prior to IFN-γ production. Yet, later on during infection, these levels declined and coincided with increased levels of anti-TLTF antibodies. Of note, it was suggested that detection of TLTF and anti-TLTF antibodies in cerebrospinal fluid of HAT patients could be used as a tool for detection and staging of the disease (141). In addition, work from the same group and Nishimura et al. (142), revealed that IFN-γ was also able to trigger TLTF secretion in *in vitro* cultures of *T. brucei* parasites in a dose and tyrosine protein kinase-dependent manner and to stimulate parasite growth (143, 144). This suggests that TLTF and IFN-γ exert bidirectional activating signals between parasites and CD8^+^ cells. Hence, these molecules might play a crucial regulatory function in the parasite–host interactions and influence the disease course during experimental African trypanosomosis (see Figure 2), whereby (i) TLTF released by *T. brucei* parasites triggers early IFN-γ production by CD8^+^ T cells leading to the activation of M1 cells, (ii) IFN-γ triggers further secretion of TLTF by the proliferating parasites and was also suggested to be a growth factor for trypanosomes (142, 145). However, an alternative explanation for the apparent IFN-γ-mediated parasite growth effects, which cannot be excluded in *in vivo* settings, is that the early expansion of proliferating parasites (cf. ascending phase of first peak parasitemia) releases more TbKHC which in turn stimulates the synthesis of the essential nutrients, i.e., polyamines (see above) (88).

#### TbTSIF Induces M1 Cells and Triggers T-Cell Suppression

Several trypanosome components have been shown to exert a macrophage-activating potential, leading to NO-dependent suppression of T-cell proliferation (146, 147). Another parasite-derived molecule exerting the potential to modulate the host immune network is the *T. brucei*-derived Trypanosome Suppression Immunomodulating Factor (TSIF) (148). Since this molecule plays a role in triggering suppressive M1, it is most likely released during the descending phase of infection (see Figure 2), at the moment that M1 cells exert their most prominent effects (i.e., production of trypanolytic molecules TNF/NO). Furthermore, as shown by Gomez-Rodriguez et al. (148), this molecule is able to (i) block T-cell proliferation in a cell–cell contact and IFN-γ/NO-dependent manner and (ii) limit secretion of immune-protective IL-10 by alternatively activated macrophages (M2) required to dampen M1-mediated pathogenic effects. Hence, TbTSIF could play a dual role, i.e., contribute to initial parasite control (via TNF/NO) and fuel suppressive M1 and T-cell suppression leading to pathogenicity. However, T-cell suppression could also be a means of the parasite to negatively affect/inhibit B-cell development and thereby impair effective humoral responses (see later) and allow/guarantee parasite survival. In addition, it seems that TbTSIF is also essential for *T. brucei* development/biology since TbTSIF knock-out parasites were not viable and died within 2 days.

### Host Innate/Adaptive Responses Determine Trypanosome-Associated Pathogenicity

Since AT can establish chronic infections in their mammalian host, which is associated with different forms of pathogenicity (anemia, liver injury, weight loss, neuropathology,…), it is clear that the innate response is insufficient for complete elimination of the parasites and, hence, will require the help of the adaptive immune response to combat infection. Yet, the modulation of the innate immune response might also affect the rejoinder of the adaptive immune response. In this section, we will elaborate on what is happening during the later stage of infection once the trypanosome infection is established in the mammalian host.

#### Trypanosome-Infections Impair B-Cell Functionality

As mentioned before, although trypanosomes use antigenic variation of their VSG coat as an efficient way to escape host humoral responses, trypanosomes also directly/indirectly affect B cell development as an additional means to escape elimination. Important to mention is that experiments in μMT (B cell deficient) and IgM^−/−^ mice revealed that the initial development of peak parasitemia is independent of infection-induced anti-VSG antibodies. In addition, *in vivo* parasite VSG switching is an intrinsically programed genetic process that is independent of B cells or antibody pressure, with the function of antibodies mainly limited to the elimination of the remaining non-switched parasites (149). Studies in experimental rodent infection models have implicated T-cell-independent anti-VSG IgM responses to be the first line of host defense against proliferating parasites (150) (see before). Although B cells aid in periodically clearing circulating parasite levels by VSG-specific antibodies, they are limited by their VSG-specificity, yet they are required for long-term survival, while IgM antibodies play only a limited role in this process (149, 151–153). Importantly, similar observations were obtained in a Cape Buffalo model for natural trypanosomosis resistance (154). An additional aspect that plays a role in antibody-mediated recognition of trypanosomes is that though polyclonal antibodies are raised against different parts of the VSG molecule (155), only surface exposed regions (N-terminal more variable region) of the VSG could play a role in parasite elimination given that the buried epitopes (C-terminals more conserved region) are inaccessible for conventional antibodies (102, 156). Indeed, the VSG coat functions as a protective coat shielding of conserved buried epitopes/proteins, thereby preventing elimination of successive waves of trypanosomes expressing a different VSG coat.

The data so far indicate that *T. brucei* parasites affect B cells already early during infection (within 1 week p.i.) at different levels, resulting in the loss of humoral immune competence in trypanosusceptible hosts. This early undermining of humoral responses is important given that the production of high-affinity, antigen-specific, class-switched, antibodies takes up to 10 days after immunization (157). First, as mentioned before, there is induction of non-specific, polyclonal B-cell activation leading to clonal exhaustion (158–160). Second, there is destruction of the splenic B cell compartment that is manifested by the occurrence of marginal zone and follicular B cell (FoB) depletion. Hereby, IFN-γ was shown to play a key role in destruction of FoBs (161), which was associated with enhanced expression of the death receptor Fas, leading to loss of protective B cell memory responses against unrelated antigens. Third, it was shown that during *T. brucei* infection there is an impaired B-cell lymphopoiesis in the bone marrow and spleen already at the level of transitional B cells (159, 162). Hereby, there was massive cell death observed in transitional B cells *in vitro* through a contact-dependent mechanism, which is not dependent on TNF or prostaglandin-dependent death pathways (159). Of note, the mechanism(s) of *T. brucei*-induced transitional B-cell depletion *in vivo* remains to be fully elucidated.

Collectively, trypanosomes deliberately undermine the host’s capacity to sustain antibody responses against recurring parasitemia waves by depleting transitional B cells, which in turn impairs the replenishment of the mature marginal zone and FoB populations. Since parasite-specific antibodies are essential for parasite control, inhibition of B-cell maturation at the transitional stage is an efficient evasive mechanism to prevent the buildup of protective “humoral” immunity against successive parasitemia waves. In this context, it was recently shown by De Trez et al. (163) that *T. brucei* infection is impairing the maintenance of the antigen-specific plasma B-cell pool.

#### Trypanosome Infections Induce Early IFN-γ-Mediated M1 Polarization that Subsequently Contributes to Pathogenicity Development

The parasite-derived components sVSG and CpG DNA that are released trigger via specific receptors (SR-A, TLR9) myeloid cell activation (121–123, 127, 164). In turn, this triggers T cell activation and the release of IFN-γ (165), which primes macrophages to become fully activated/M1 polarized thereby releasing pro-inflammatory molecules (TNF/NO) needed for parasite control (166, 167). This type 1 cytokine storm can also culminate in pathology development if maintained during later stages of infection (166–172). Yet, only animals able to produce tissue-protective IL-10 can exhibit an alleviated pathogenicity (167). Importantly, the balance of these different activation/deactivation signals may determine the outcome of infection (173, 174). Recently, it was shown that different lymphocyte populations play a role in IFN-γ production, whereby NK and NKT cells are the earliest IFN-γ producers, followed by CD8^+^ and CD4^+^ T cells (124). A possible explanation for this transition in different IFN-γ-producing T cells during the early stages of infection could be that: (i) initially type-I IFN released by for instance TLR9-activated myeloid cells can trigger NK/NKT-cell activation (175, 176); (ii) subsequently, parasite-derived TLTF will trigger IFN-γ production by CD8^+^ T cells in a non-antigen-specific manner (140, 145, 177); and (iii) finally, the increased release and subsequent processing of sVSG will lead to MHC-II presentation and activation of CD4^+^ T cells, thereby further fueling IFN-γ production and M1 polarization (178).

Whatever the source of IFN-γ may be, research so far indicates that early IFN-γ production triggers an acute inflammatory reaction resulting in acute anemia development, as witnessed by a 50% reduction in circulating red blood cells (RBC) within 2 days following peak parasitemia. After a short recovery phase, a subsequent gradually increasing loss of RBCs occurs during the chronic infection stage (166, 167, 179). Of note, anemia development was found to be independent of antibodies and the height of the parasitemia peak, whereby the acute nature of this phenomenon implies a consumptive etiology (149, 168). IFN-γ plays also a crucial role in the recruitment and activation of erythrophagocytic myeloid cells. In addition, the work of Cnops et al. (124) indicates that the absence of NK, NKT, and CD8^+^ T cells, but not CD4^+^ T cells, during the early stage of infection results in a reduced anemic phenotype similar to IFNγR−/− mice. In addition, it was recently shown that trypanosomes can release extracellular vesicles (EV) that can fuse to mammalian erythrocytes thereby changing their physical properties and making them more susceptible to erythrophagocytosis (86). This in turn leads to acute anemia and could be a means of the parasites to acquire essential nutrients [hemoglobin and/or iron (see before)]. Hence, both host-induced and parasite-induced factors could account for acute anemia development. Subsequently, the hosts’ ability to respond to the acute anemia will determine whether anemia persists or not during the chronic phase of infection (55, 166, 180–182).

Another pathological feature associated with *T. brucei* infections is neuropathology, whereby parasites pass the BBB and cause severe neurological complications. Interestingly, the work of Amin et al. (183) showed that *T. b. brucei* parasites penetrate the BBB very early during infection (within 2–3 days post infection), whereby they proposed that TLR9 and MyD88-mediated activation of DCs triggers via type-I IFN (IFN-α/β) T-cell activation. Subsequently, these activated T cells invade the central nervous system (CNS) in a IFN-α/β, IFN-γ and TNF-dependent manner, whereby TNF can induce the expression of adhesion molecules (ICAM-1 and VCAM-1) in brain endothelial cells in a TNFR1-dependent manner and contributes to the leakiness of inter-endothelial cell tight junctions or stimulation of matrix metalloproteases activities that open the parenchymal basement membranes (184, 185). Furthermore, the same group showed that IFN-γ, as well as the IFN-inducible chemokine CXCL10, promotes the penetration of T cells and parasites in the brain (186, 187), suggesting that parasites can also follow T cells during their brain invasion across the BBB. However, the work of Frevert et al. (188) showed, using a murine model and intravital brain imaging, that bloodstream forms of *T. b. brucei* and *T. b. rhodesiense* enter the brain parenchyma within hours post injection, before a significant level of microvascular inflammation is detectable. Yet, there are differences in the trypanosome strain used and the infection dose as well as the route of infection that could account for the different results. Collectively, it seems that whatever mechanism (host-mediated or not) parasites use to pass the BBB and infiltrate the brain, extravasations of parasites from the blood into the brain might be an alternative evasion mechanism to escape humoral responses that predominate in the blood circulation and thereby allow future transmission when parasites migrate back into the blood.

## Conclusion and Perspectives

Overall, it seems that trypanosomes have evolved efficient immune escape mechanisms to sculpture the hosts’ innate/adaptive immune response in order to establish an environment suitable for parasite survival and transmission. This manipulation of the host response has its cost since this undermines the hosts’ capacity to respond/recover following establishment of the parasites. Hereby, persistence of inflammation during the chronic stage of infection culminates into pathogenicity and subsequent death if left untreated. Hence, identification of host-derived factors playing a role in persistence of inflammation could be an alternative means to alleviate trypanosomosis-associated pathogenicity. In this context, it was recently shown that the pleiotropic host molecule macrophage migration inhibitory factor (MIF) plays a key role persistence of inflammation and infection-associated pathogenicity (180). Hence, future intervention strategies against African trypanosomosis might require a dual approach, i.e., development of efficient anti-trypanosomal agents combined with neutralization of anti-pathogenicity inducing “host” factors, which combined might allow reducing the economical losses of the affected continents.

## Author Contributions

BS, GC, JA, JG, SM, and CT wrote the manuscript.

## Conflict of Interest Statement

The authors declare that the research was conducted in the absence of any commercial or financial relationships that could be construed as a potential conflict of interest.

## Abbreviations

BBB, blood–brain barrier; DMG, dimyristoyl glycerol; GPI, glycosylphosphatidylinositol; Hb, hemoglobin; Hp, haptoglobin; HpHbR, haptoglobin–hemoglobin receptor; LS, long slender; M1 cells, classically activated myeloid cells; NHS, normal human serum; SR-A, scavenger receptor type A; SRA, serum resistance antigen; SS, short stumpy; TbAdC, *T. brucei* adenylate cyclase; TbKHC, *T. brucei* kinesin heavy chain; TgsGP, *T. b. gambiense*-specific glycoprotein; TLF, Trypanosome lytic factor; TLTF, T-lymphocyte-triggering factor; TSIF, trypanosome suppression immunomodulating factor.
